# Translating evidence-based knowledge objects into practice

**DOI:** 10.3389/frhs.2023.1107096

**Published:** 2023-09-11

**Authors:** John Damm Scheuer

**Affiliations:** Department of Social Sciences and Business, Roskilde University, Roskilde, Denmark

**Keywords:** translation, knowledge translation, organizational translation, implementation, Cochrane review, clinical guideline, translation theories, translation models

## Abstract

This paper aims to show how organizational translation theories and models may supplement implementation science with a new process perspective on how knowledge objects such as Cochrane reviews, clinical guidelines and reference programs are implemented in practice in healthcare organizations. They build on Bruno Latour's idea about translation that states that the spread in time and space of anything—including knowledge objects—is in the hands of people and that each of these people may act in many different ways, letting the token drop, modifying it, deflecting it, betraying it, adding to it, or appropriating it. Implementation science theories, models and frameworks often try to identify general aspects of processes and variables that influence implementation processes. In contrast, translation theories and models build on a process view that uses the sequence of events, activities and choices by translators situated in time as well as in space to explain how outcomes of translation/implementation processes came about. The paper develops some implementation relevant propositions about translation of knowledge objects in healthcare organizations that may inform further research. Moreover, it discusses how organizational translation studies and implementation science may supplement each other.

## 1. Introduction

A knowledge object is a piece of knowledge held in a well-defined and structured format, such that it is easy to replicate and disseminate. It typically contains explicit evidence-based knowledge but may also contain some elements of human knowledge (KM Glossary, skyrme.com). Examples of knowledge objects in healthcare organizations include Cochrane reviews, reference programs and clinical guidelines. Generally, knowledge objects in healthcare organizations are intended to inform practitioners about the latest evidence-based knowledge related to certain types of patients and diagnoses and to support and improve their decision-making concerning these patients. They contain an assembly of evidence-based knowledge and ideas about “what to do” with certain types or categories of patients. In order to assure that evidence- based knowledge objects have an impact on practice, they need to be implemented or, as assumed in this article, “translated”.

**Table 1:** Selected translation models.

Theories	Linguistics	Symbolic interactionism	Actor-network-theory Ventriloquism^a^	Neo-institutional theory
Models	Holden et al.'s Knowledge translation model	Carliles’ knowledge translation model	The idea-practice-translation model	The travel of ideas model
Authors	Holden et al. (7)	Carlile (6)	Scheuer (3)	Czarniawska and Joerges (5)

^a^ Ventriloquism was developed by the organization studies researcher Cooren (8). He based his theory about the communicative constitution of organizations partly on actor-network-theory. The idea-practice-translation model builds on its ontological and epistemological assumptions about organizations.

The translation perspective on organizational change has developed in organization studies in recent years among researchers who study the movement of management and organizing ideas as well as other tokens in organizations (1–3). It focuses on understanding how different types of ideas/tokens move within as well as between organizations. The types of tokens that organizational translation researchers have studied include the translation of new management and healthcare ideas, of strategies, policy ideas, the movement of knowledge, translation in relation to socio-technical co-construction and design of IT-systems as well as in relation to the creation and translation of ideas and knowledge during innovation processes (3). This article will focus on what implementation science researchers may learn about implementation processes related to knowledge objects from the theories and models of translation that have been developed in organization studies (1–3). It offers a new view on implementation as translation processes that may supplement and—if further researched—develop especially the process dimension of existing frameworks in implementation science.

So, what is translation in organization studies? Many different definitions exist. One of the most famous ones suggests that

“*….the spread in time and space of anything—claims, orders, artefacts, goods—is in the hands of people: each of these people may act in many different ways, letting the token drop, or modifying it, or deflecting it, or betraying it, or adding to it, or appropriating it.*” (4)

Latour's (4) definition suggests that the fate of any token, an idea, a concept, a knowledge object like an evidence-based reference program, a clinical guideline, or a systematic Cochrane review, depends on what the people who move them choose to do with them. They may choose to be loyal to the token or they may choose to drop the token, modify, deflect, betray, add something to it, or appropriate it. This view on organizational translation processes suggests that tokens, including those mentioned above, move geographically- that is physically—from one place to another, they move semiotically—that is in relation to what these tokens mean—and they move politically as receivers of the tokens may have interests that affect what they choose to do with the tokens (2). Therefore, in an organizational translation perspective you will expect that:

1. The translation of tokens unfolds through an uninterrupted translation chain where the token that you want to implement needs to be continuously given new energy and moved by people in a chain of translations to be implemented.
2. That the token will be adjusted and changed through the translation process because the token and what counts as knowledge in relation to it will not just be transferred but also translated and politically negotiated as it moves.

The implementation of a token in healthcare organizations—for instance an evidence-based knowledge object as a reference program, a clinical guideline, or a systematic Cochrane review—will thus demand that people and according to some translation researchers also material/physical objects are mobilized and influenced to “act” on behalf of the token (4–6). To make people and objects “act” on behalf of and through those actions in practice “realize” a token is, however, not easy. It depends on and requires that a lot of different and typically locally unique types of translation work are done before a token may be “implemented”. Giving an overview of all the insights that organizational translation studies may offer implementation science researchers is not possible in a short article. Readers interested in that may explore these issues further in Scheuer (3). Instead, the article will focus on answering the following research questions:

1. What are the implications of selected organizational translation theories and models for processes related to implementation of evidence-based knowledge objects in healthcare organizations?
2. Which conditional propositions about translation of knowledge objects may be derived from them?

The theories and models that will be discussed in the article are selected in order to demonstrate some key questions that organizational translation theories and models raise, that may interest implementation science researchers and give some new views on what may characterize implementation processes (see Table 1). The selected theories and models address questions that have been identified as important for the translation of management, organizing ideas and knowledge by organizational translation researchers that may have important implications for implementation researchers, too. Some conditioned propositions are developed on the basis of these theories and models that may inform further research of implementation science researchers. A conditional proposition consists of two simple statements joined by the words “if” and “then” (if today is Friday, then tomorrow is Saturday). A conditional proposition asserts that the antecedent implies the consequent that is: the consequent is true if the antecedent is true (9).

The implementation relevant questions that are derived from the selected organizational theories and translation models and on the basis of which the conditional propositions presented in the article are developed are: (1) What are the consequences if the knowledge object (for instance a Cochrane review or a reference programme) is considered a text that a translator needs to translate to the receivers of it? (2) What if humans/groups of humans do not just transfer knowledge/the knowledge object but also translate and politically negotiate it? (3) What if not just humans but also physical objects (non-humans) are needed to do work to implement the knowledge object? (4) May the travel of the knowledge object from one time-space context to another influence the translation of it?

In the first section of the article, the theme and research questions are presented and the concept of knowledge object is defined. In the second section, the concepts of theory and models as well as the concept of conditional propositions are explained and defined. Moreover, the phenomena and implementation situations the selected translation theories and models relate to as well as the process and inclusion and exclusion criteria used for selecting theories and models are presented. In the third section, the selected translation theories and models are presented and discussed, and some implications and conditional propositions are suggested on that basis. In the fourth section some reflections concerning the contributions of organizational translation studies to implementation science (and vice versa) are presented. Finally, in the fifth section, some conclusions are drawn.

### 1.2. Knowledge objects in healthcare

A knowledge object is a piece of knowledge held in a well-defined and structured format, such that it is easy to replicate and disseminate. Although they contain predominantly explicit (often evidence-based) knowledge, they may also contain some elements of human knowledge (KM Glossary, skyrme.com). You find many types of knowledge objects in healthcare organizations, systematic Cochrane reviews summarizing the latest evidence related to treating certain health conditions, evidence-based reference programs and clinical guidelines. Cochrane reviews attempt to collate all empirical evidence that fits pre-specified eligibility criteria in order to answer a specific research question. It uses explicit, systematic methods that are selected with a view to minimizing bias, thus providing more reliable findings from which conclusions can be drawn and decisions made (10, 11) (1.2.2 What is a systematic review? (cochrane.org)). In Denmark evidence-based reference programs are presented as a way to search for, summarize and translate scientific research results to systematic recommendations (sst.dk). According to The Institute of Medicine, clinical guidelines are “systematically developed statements to assist practitioners and patient decisions about appropriate health care for specific clinical circumstances” (12). Knowledge objects in healthcare are thus intended to inform practitioners about the latest evidence-based knowledge related to certain types of patients and diagnoses and to support and improve their decision-making concerning these patients. You may suggest that the use of knowledge objects to diffuse evidence-based knowledge to practitioners builds and depends on at least two assumptions: (1) Research-based knowledge may be stored in physical objects/texts which may then be transferred and reproduced by others/the receivers in an objective and thus non-subjective way. (2) The content of the knowledge objects may be transferred from the sender to the receiver and may be implemented without being changed by the activities and processes of the actors involved in the movement of the knowledge objects. As it will be demonstrated, the translation perspective in organization studies questions these assumptions.

## 2. Introduction to theories and models

### 2.1. From theories and models to conditional propositions

A theory may be defined as an explanation of relationships among concepts or events within a set of boundary conditions (9). A theory simplifies and explains a complex real-world phenomenon and describes the who, what and where of a phenomenon being investigated, but also explains the how, when and why it occurs (13). They consist of terms (concepts, constructs, variables, or events), relationships among terms (propositions and hypothesis) and assumptions (boundary conditions within which these relationships hold in time, space, and value contexts), and explanations (arguments that provide reasons for the expected relationships) (9). The primary phenomenon of interest for organization theorists and researchers are organizations, which includes different kinds of organizations as well as organizing activities and processes (14). Historically, many theories about organizations have developed that have then been used to develop ideas about how to change them in organization studies. As a consequence, many theories about organizations and typologies of change strategies and models based on them have been developed in organization studies (15, 38, 39)^1^. This approach has also characterized organizational translation studies and organizational researchers' attempts to theorize and model change processes in organizations as translation processes (3).

As pointed out by Nilsen (40), models involve a deliberate simplification of a phenomenon or aspect of a phenomenon and need not be completely accurate representations of reality to have value (41, 42). Morrison and Morgan (43) argue that models serve as mediators between theories and data. They may not be derived entirely from theory or from data because they are neither one thing nor the other, neither just theory nor data, but typically involve some of both (and often additional “outside elements”), so that they can mediate between theory and the world (43). The organization researcher McKelvey (44) thus suggests that social scientists do not directly observe or test theories; instead, they examine models, and models may be seen as partial representations or maps of theories. Therefore—as pointed out by Nilsen (40)—models are closely related to theory and the difference between a theory and a model is not always clear.

When referring to translation “theories and models” in this article, it refers to the above-mentioned definitions and understandings of these concepts. Several of the approaches to translation and organizational change that have been included in this paper are embedded within and draw upon the basic assumptions of well-known and accepted theories in organization studies like institutional theory (the idea model) (5) and symbolic interactionism (6), Actor-network Theory (4, 45, 46) and Ventriloquism (the idea-practice-translation model) (8, 47). Other theories and models draw upon linguistic theories that model translation processes as characterized by translation of texts (which may be both written and/or spoken by the translators) (7). An overview of the theories and models included is shown in Table 1.

**Figure 1:**
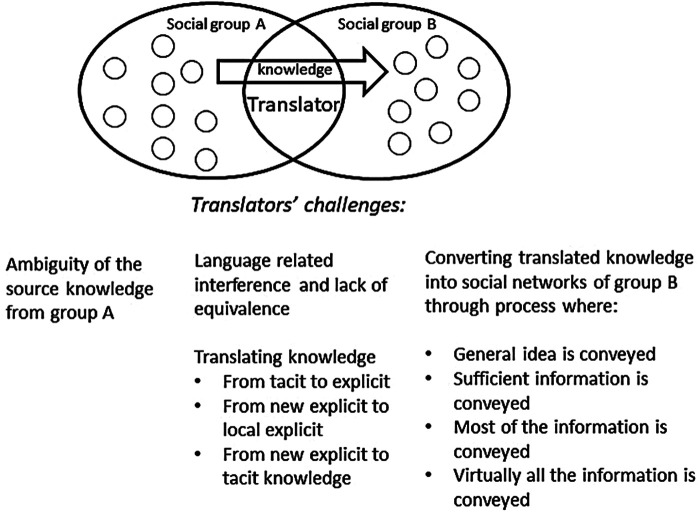
Extended model of knowledge transfer as translation. Source: developed by the author from Holden et al. (7).

The selected theories and models referred to in the article are used to formulate a number of conditional propositions concerning implementation processes. A conditional proposition consists of two simple statements joined by the words “if” and “then” (if today is Friday, then tomorrow is Saturday). A conditional proposition asserts that the antecedent implies the consequent that is: the consequent is true if the antecedent is true (9). In the article, some selected theories and models are referred to that organizational translation researchers suggest identify some key characteristics of the way management ideas, knowledge and other tokens have been translated in organizations. In this article, some conditional propositions are deduced from them and it is suggested that if these (the above-mentioned) propositions are relevant to the translation of management ideas, knowledge and other tokens in organizations, then they might be relevant to implementation of knowledge objects in healthcare organizations, too. Here it should be noticed that propositions and hypotheses differ by levels of abstraction: propositions are relationships among theoretical concepts or constructs, while hypotheses are relationships among concrete observable variables or events (9). Thus, in order to test the relevance of the conditional propositions put forward in this article for implementation processes, they need to be translated into hypotheses, observable variables and events and tested empirically in later studies.

The selected theories and models theorize and model the translation process differently and are based on different ontological assumptions (1–3). In organizational translation studies, this has made some researchers discuss whether these issues might suggest that the theories and models focus on different phenomena and belong to separate and perhaps incompatible research traditions (2, 3). They conclude, however, that they do not believe this to be the case. Instead, they suggest that the theories and models focus on different aspects of translation processes, and do so with different emphasis and terminology. They moreover conclude that they are complementary and try to say something about the same phenomenon: How an object changes from one state to another within and across organizational settings (2, 3).

### 2.2. Inclusion and exclusion criteria

The inclusion criteria used when selecting the theories and models were their ability to demonstrate some questions that organizational translation theories and models have identified and raise, which may interest implementation science researchers and give some new views on what may characterize implementation processes and make implementation of evidence-based knowledge objects difficult (these questions are described in the introduction). They represent different views on what may affect the translation of a token like a management concept or idea or as hypothesized here; a knowledge object as it moves through translation chains of people and/or groups of people in or between organizations. Translation theories and models thus offer a process view (48) on organizational change and implementation that may be considered an alternative to existing process views in implementation science (see section 4 below for a discussion of this).

The selection of theories and models was based on an in-depth literature review of organizational translation theories and models that was performed by the author when writing his latest book: How Ideas Move—Theories and Models of Translation in Organizations, Routledge (3). The research for the book started out from existing reviews of the research literature in organizational translation studies including reviews by O'Mahoney, Scheuer, Wæraas and Nielsen and Wedlin & Sahlin (1, 2, 49, 50). These reviews were supplemented with an additional literature review conducted especially to support the research done when writing the book. In this literature review, the most cited theories and models in different areas of organizational translation studies were identified as well as theories/models that represented different definitions and understandings of translation and the translation process in organization studies.

Concerning the exclusion criteria, some organizational translation theories and models were excluded from the article due to lack of space or relevance [an overview of other translation theories and models in organization studies may be found in Scheuer (3)]. Another research stream that was excluded was linguistic studies of the translation of texts—primarily those focusing on the translation of texts (books, instructions, user manuals, etc.) from one language to another rather than on translation of tokens between groups of people in organizations aimed at being implemented and causing organizational change. The linguistic theory about knowledge translation in organizations that was included in the article (7), thus has an explicit focus on translation of texts and knowledge aimed at being implemented and causing organizational change.

### 2.3. What do the selected theories address and in which situations are they relevant?

The selected translation theories and models presented in this article build on the assumptions that were mentioned in the introduction and try to theorize and model how translation processes unfold in different situations. Each of the selected theories and models focus on phenomena that may make the movement (and thus implementation) of knowledge objects difficult in healthcare organizations. The theories and models that have been selected address:

- The consequences of viewing translation of knowledge objects as a linguistic translation of texts that may include good, bad, wrong translations and depend on translators' translation competences (7).
- Translation processes as characterized by not just transfer but also intergroup translation and negotiation of the content and knowledge related to the knowledge object (6).
- Translation processes as dependent on both humans and physical objects' (non-humans') work and thus—according to some translation researchers—complex, locally situated socio-technical design and translation processes (3).
- The travel and physical disembedding, re-embedding and translation of knowledge objects from one time-space context to another that may be caused by rational human actors trying to make their organizations more effective and efficient but is often also caused by other things: What translators happen to attend to, characteristics of the knowledge objects themselves, normative pressures and influence from fashion trends (5, 51–53).

Each of the above-mentioned theories and models focus on different situations where an evidence-based knowledge object needs to be translated in order to be moved and thus implemented—and where some difficulties may arise in order to succeed with such an endeavour. These situations include situations where:

• Translators of an evidence-based knowledge object (a Cochrane review, a reference programme, a clinical guideline) try to translate the knowledge object in the form of a document or a text to practice in their local organization/department/unit.
• Situations where different groups having different cultures and languages try to transfer, translate, and negotiate what should count as knowledge in relation to the knowledge object at encounters between the groups.
• Situations where not only humans but also “non-humans” i.e., material objects of different sorts need to be included in the translation process in order to succeed with implementation (as when you develop and introduce diabetes monitoring IT-systems at hospitals which makes both humans and IT-systems an object of design efforts)
• Situations where local, socially embedded translators in healthcare search for and may direct their attention toward relevant evidence-based knowledge objects (as many health care scientists and practitioners think they are supposed to) but may also just as well direct their attention elsewhere when trying to identify solutions to their local problems.

## 3. Translation theories and models

### 3.1. Translation as translation of text objects

As pointed out by Malmkjær (54) linguistics is the academic discipline that focuses on languages, and translation can be seen, in Catford's (55) words as “an operation performed on languages”, and as pointed out by the knowledge translation researcher Holden (7) “translation…is by far the oldest universal practice of conscientiously converting knowledge from one domain (i.e., a language group) to another”. Holden et al. (7) thus point out that human languages differ in relation to their syntax (the way in which words are arranged and combined grammatically), in their morphology (that is in how they are used in certain contexts), in their lexis (which refers to the vocabulary items of a language) and in their phonology (which refers to the speech sounds of a language). He moreover points out that these four elements deviate from each other among languages and that language may be seen as a repository of knowledge, experience and impressions and a device for facilitating social interaction. The challenge of the translator in finding equivalence as he/she translates between groups is then not just to render the words of one language into a second one, but also to re-express psychological and related factors within the terms of reference of that second language (7). They therefore conclude that:

• Knowledge transfer in organizations, like literary translation, is a sense-making activity.
• Knowledge transfer, like translation, is literally concerned with personal cognition and the inter-lingual transfer of knowledge from head-to-head and into social networks.
• Knowledge transfer, like translation, is subject to constraints, which affect not just transfer, but rather transferability: the extent to which knowledge can be transmitted to others.

As summarized by Scheuer (3), Holden et al.'s (7) model (see Figure 1) theorizes the factors that influence knowledge translation processes when knowledge moves between cross-cultural teams [see (7)]. The first factor is the lack of cultural understanding, uncertainty and thus ambiguity related to the source of the knowledge that leaves room for interpretation by the receiving group or team. Other factors are interference and lack of equivalence, which refers to the errors of translation that may occur because of differences in the use of words, grammar or pronunciation between the source and target language and the (possible) lack of corresponding words and concepts between the languages of the sender and the receivers. Other factors that influence the knowledge translation process are:

1. The ability of the translators or receivers of new knowledge to make tacit knowledge that is necessary for the functioning of the knowledge and is acquired through socialization explicit,
2. The translators' and receivers' ability to combine new and local explicit knowledge in relevant ways and
3. Their ability to internalize and make this new explicit knowledge tacit again.

Moreover, the knowledge translation process is influenced by the translatability and convertibility of the knowledge that is being translated. The translatability of the knowledge concerns the properties of the knowledge and whether the translator is a domain expert both in terms of the languages between which he needs to translate and in terms of the subject matter of the text/knowledge being translated. The convertibility of the knowledge will depend on whether domain experts/translators as well as other receiving team/group members find it useful and choose to implement it. Finally, when the knowledge has been through this process the translated knowledge may be converted into social networks (the receiving teams/groups) in at least 4 different ways: (1) The general idea is conveyed, (2) sufficient information is conveyed, (3) most of the information is conveyed and finally, (4) virtually all the information is conveyed.

#### 3.1.1. Implications and conditioned propositions

The implications of what was mentioned above for implementation of text-based knowledge objects (like Cochrane reviews or guidelines) are that those who receive them need to have very similar cultural backgrounds and use language in very similar ways for the knowledge object to make as much sense to them as it did to the senders of it. Moreover, those who implement (translate) it need to be able to identify and handle both explicit and tacit aspects of the knowledge object that are necessary to “make it work” in the receiving group and if it doesn’t; to improvise in a way that ensures that it does. Finally, both characteristics of the knowledge object itself, its usefulness for the receivers and how information about it is communicated may play a role.

The conditioned propositions that may be derived from what was mentioned above concerning implementation of evidence-based knowledge objects in healthcare organizations are that: (1) Implementation of knowledge objects as text-objects may need to be translated from one language and cultural group to another as it moves through and between groups of people in healthcare organizations, (2) this movement may depend on the types of language, learning and culturally related factors that Holden et al. (7) point out, (3) the implementation and adaptation of an evidence-based knowledge object may be studied by researching how it is translated and put into (written and spoken) words by different people and groups of people as it moves through the translation chain and, (4) effects related to the knowledge object may be assumed to relate to how this is done.

### 3.2. Translation as intergroup transfer, translation, and negotiation

Some researchers in organizational translation studies build on the ideas of symbolic interactionism (56). In this view, humans' capacity for thought is shaped by social interaction. It is assumed that people learn the meanings and the symbols that allow them to interpret and act in meaningful ways in different situations through their actions as well as interactions with other humans. People and groups examine possible courses of action related to a situation, assess their relative advantages and disadvantages, and then choose one that seems appropriate given the situation at hand (57). As a consequence of this view, some translation studies researchers theorize organizational translation processes as an intergroup transfer, translation and negotiation process (6, 58, 59). Translation is theorized as happening between people and groups of people belonging to different social worlds (56). These social worlds are often remote from each other culturally, language-wise, in relation to interests as well as in time and space. This now creates problems whenever collaboration and coordination of several groups of people are needed in order to achieve common social goals (as for instance when “implementing” an evidence-based knowledge object).

**Figure 2:**
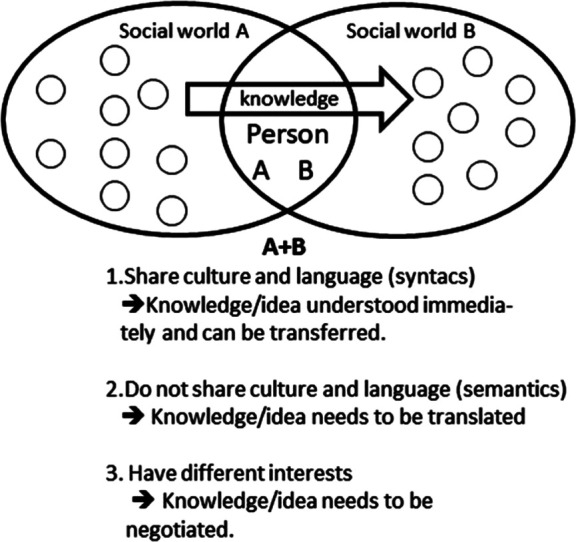
Carliles knowledge translation model. Source: developed by the author from Carlile (6).

As explained by Scheuer (3) the knowledge translation model of Carlile (6, 59) (see Figure 2) suggests that if social worlds A and B are similar in their language, culture and interests, knowledge about (for instance) a knowledge object may just be **transferred**. The knowledge will be relatively easy to communicate and will be relatively easily accepted by the receivers and storage and retrieval technologies may be used to store the knowledge for later use. If there is a greater distance between the two groups in their language, culture, and interests, however, the knowledge (object) also needs to be translated and politically negotiated.

The reason is that if something new is created or presented during an innovation or translation process, it is not sufficient to share and assess knowledge across a boundary. In that situation, a new situation arises that creates a semantic boundary that necessitates a **translation** or interpretive approach. Novelty thus generates some differences and dependencies that are unclear—different interpretations exist. Common meanings are developed to create shared meanings and provide an adequate means of sharing and assessing knowledge at the boundary. In that situation, the different communities of practice engage in translating knowledge in order to create shared meanings. During this process, the techniques used are development of the different groups' semantic capacity, cross-functional interactions, and teams as well as boundary spanners and translators and according to some translation researchers also “boundary objects” (58).

Carlile (6, 59) points out that being able to create common meanings and to be able to share and assess knowledge, you often need to take differences in interests between members of group A and group B into account and make new political agreements. Novelty thus potentially generates different interests between actors that impede their ability to share and assess knowledge. Common interests are therefore developed to transform knowledge and interests and provide an adequate means of sharing and assessing knowledge at a boundary. Knowledge is therefore not just translated but also negotiated and through that political process **transformed**. The techniques required by actors involve an ability to be pragmatic, to use prototyping and other kinds of boundary objects that can be jointly transformed. To share and assess knowledge thus requires significant practical and political effort.

Finally, Carlile points out that several iterations are needed. Addressing the consequences of knowledge (a knowledge object) cannot be resolved by group A and B with one try but requires an iterative process of sharing and assessing knowledge, creating new agreements, and making changes where needed. As the actors participate in each iterative stage, they get better at identifying what differences and dependencies are of consequence at the boundary; they improve at collectively developing a more adequate common lexicon, meanings, and interests (6).

#### 3.2.1. Implications and conditional propositions

The implications of Carlile's (6) knowledge translation model for implementation of evidence-based knowledge objects in healthcare organizations are that a knowledge object will be easier to transfer if the receivers of it shares the language, culture and interests of the senders of the knowledge object in question. If they do not, however, more translation and negotiation work will probably be needed. Another implication is that if more groups along a translation chain are involved, even more translation and negotiation work is needed, and even more uncertainty may be introduced in relation to how the knowledge object is translated. As a consequence, it may be assumed that it is more likely that the knowledge object/the knowledge it communicates will be changed as it moves through these groups than it will remain the same.

The conditional propositions that may be formulated concerning the implementation of evidence-based knowledge objects in healthcare organizations based on this are: (1) An evidence-based knowledge object (the knowledge it represents) may just be transferred if the groups involved in the process are alike language-wise and in their culture and interests, (2) it will have to be translated and politically negotiated if it is not, (3) the more different groups of people in the translation chain, the more translation and negotiation work needs to be done, (4) the degree to which the original content of the knowledge object is preserved or changed through the process may be considered uncertain and an empirical question.

### 3.3. Translation processes as dependent on both humans' and non-humans' work

After having focused primarily on humans' work organization and science and technology studies researchers have increasingly recognized the importance of the work that physical objects and things (materialities) do in organizations. Thus, after having been ignored for many years, actor-network theory and science and technology (4, 45, 60, 61), process-study (62), learning (63) and communication researchers (47) in organization studies have accepted that both humans' and non-humans' (objects/things/materials) work is important in organizing processes^2^.

A Cochrane review of Arthroplasties (with and without bone cement) for proximal femoral fractures in adults (64) found that there is good evidence that cementing the prostheses in place will reduce post-operative pain and lead to better mobility. It points out some work that doctors (humans) in a department need to do to “implement” or rather translate the knowledge object; they should choose solutions where the prosthesis is cemented in place instead of other solutions. It also points out some work that non-humans seem to do more or less well in these situations (different artificial joints that doctors may choose from that include different shapes of the stem set into the bone; the incorporation of a secondary joint (bipolar joint); joints that replace only the ball part of the ball and socket hip joint (hemiarthroplasty) and those that also involve replacing the socket part of the hip joint (total hip replacement). As a consequence, to “implement” or rather to translate the above-mentioned knowledge object, translators receiving the Cochrane review in a local orthopedic surgery department need to design and construct new relations and types of interactions between both humans (the patients having hip problems, the doctors who perform hip operations in the department) and non-humans (the different types of prostheses available for such operations) in order to translate the knowledge object. The (performative) effects of the knowledge object will depend on whether a translator succeeds with this local translation and construction of new types of relations and interactions between humans and non-humans (artifacts/things/objects).

Scheuer's (3) “idea-practice translation model” was developed to theorize and model what happens in the encounter between a translator wanting to translate an innovative token (as an idea about a diabetes monitoring system or a knowledge object full of ideas about what to do with certain patients etc.) and a local context as it is translated. It is based on research-based insights from organizational research in actor-network theory (4, 45, 46), ventriloquist communication (8, 47), learning (65, 66) and design processes (67) as well as from research in organizational routines (68) and relational inertia (3, 69). It suggests that change processes in hospitals and other types of organizations are socio-technical (or socio-material) design and construction processes^3^. Scheuer (3) suggests that translation processes that “materialize” (and thus implement) tokens such as evidence-based knowledge objects have the following characteristics:

1. The token (knowledge object) has to be translated into an “actor-network” of humans and non-humans (objects/things/materials) that then do the work that materializes and thus “implements” it (4, 45, 46).
2. The organizing of the humans and non-humans necessary to translate the token (knowledge object) depends on communication and dialogues. In these (ventriloquist) dialogues (8, 47) translators may communicate that they think that certain humans and non-humans are necessary for translating the token. But also unexpected humans and non-humans may communicate and make the translators speak and act in certain ways in that connection (as when surgeons will not change their routines for some reason, or the cement used to cement a prosthesis in place does not fasten it enough and translators need to communicate and try to do something about both problems)
3. The translation process moreover depends on a socio-technical design process. It includes designing and constructing new relations and types of interactions between humans, objects, and contexts (as demonstrated in the example above) (67).
4. The translation process also depends on translators learning which humans, non-humans (objects/artifacts) and contextual factors are relevant for the translation and materialization of the token (the knowledge object) in a given translation situation. Here learning may originate from translators' interaction with locally present “body-external” humans, non-humans and contextual factors. Furthermore, it may originate from the translators' “embodied/internal” reflections about his/her former experiences from interacting with similar types of humans and non-humans in similar contexts, about his/her idea about the future goal of the process or about his/her own understanding of own identity and feelings (65, 70).
5. To be implemented the token (knowledge object) moreover has to be translated into new relations and interactions between humans and non-humans that are then stabilized (and thus become reproduced continuously across time). The stabilizing happens through a process where the translators connect an assembly of certain humans and non-humans, certain activities/actions and supporting artifacts with a narrative about the assembly that makes sense to the translators (68) (“in our department the operation of Arthroplasties for proximal femoral fractures in adults should involve these actors, who interact in this way following these procedures, using these prostheses based on these reasons” etc.).
6. Both symbolic and socio-material tools may be developed and used by the translators to translate the token (knowledge object) (65, 66). Symbolic tools **may be the Cochrane review mentioned above**, theories, models, calculations, or preliminary interpretations about how to design and construct the relevant assembly of humans and non-humans (objects/materials). Socio-material tools may include local experiments and development of prototypes where different assemblies of humans, their activities/types of actions, objects, and narratives about the token are tried out in practice.
7. Finally, the translation process (and thus the implementation of the token/knowledge object) depends on whether the relational inertia (3, 69) that hinders the translation of the token is overcome. Relational inertia is produced by humans and/or non-humans not relating and interacting in the way they need or are supposed to if the token (knowledge object) is to be materialized realized/implemented). Overcoming relational inertia therefore depends on translators' ability to somehow—through appropriate strategies—solve the conflicts and controversies with all these humans and/or non-humans.

**Figure 3:**
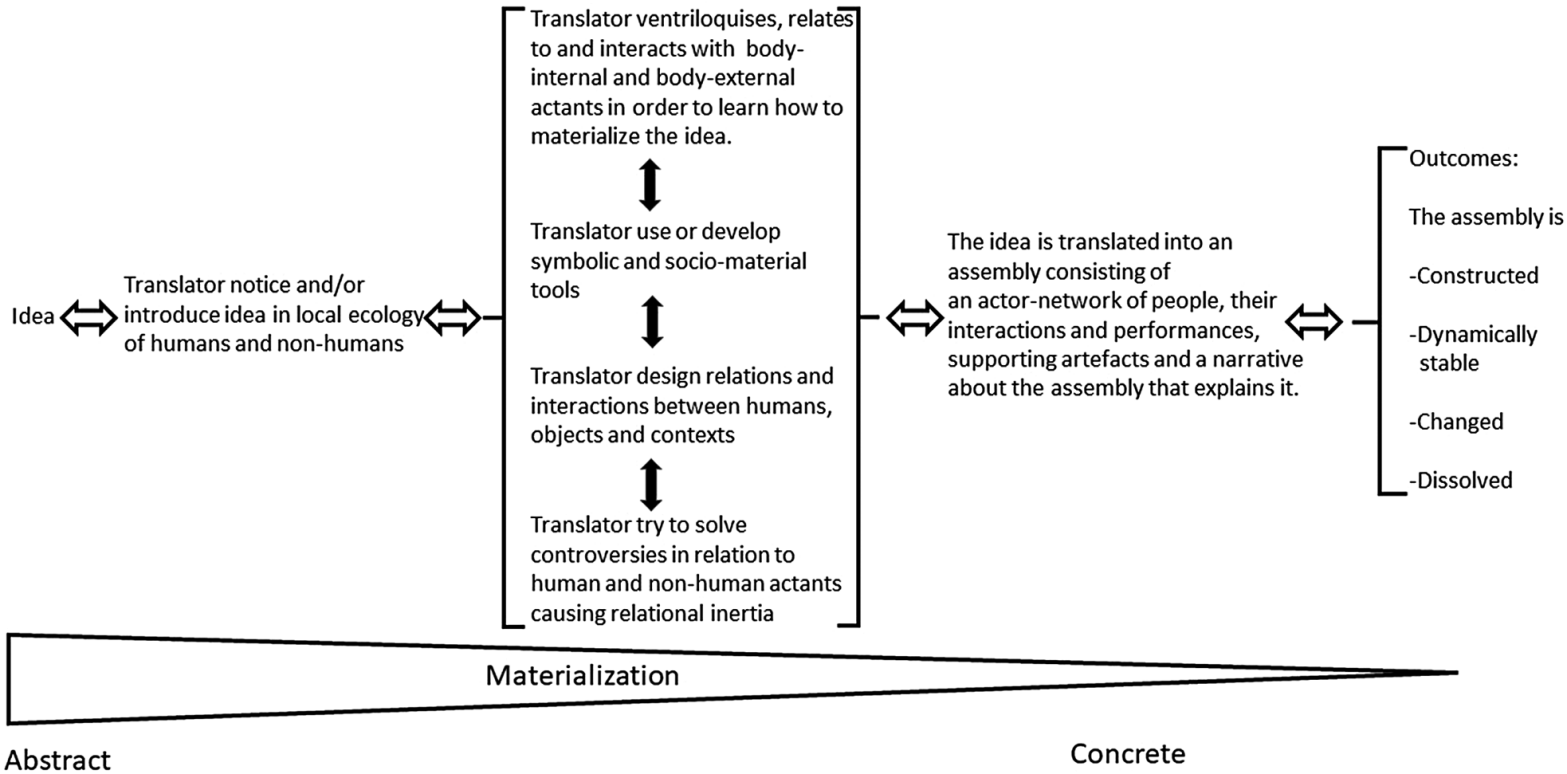
The idea-practice translation model. Source: Scheuer (3).

The idea-practice translation model (3) (see Figure 3) builds on these assumptions and suggests that the translation of a token (as a knowledge object) will unfold as follows:

**Figure 4:**
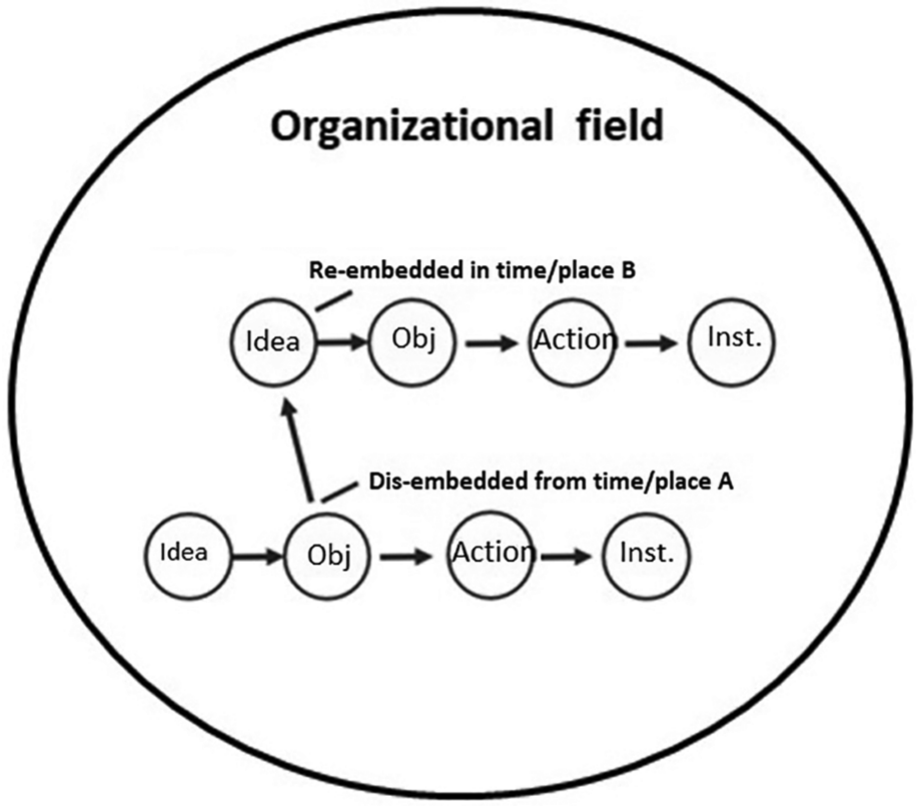
The travel of idea model. Source: developed by the author from Czarniawska and Joerges (5).

Innovative ideas (as those related to knowledge objects) are first noticed and introduced by the translator(s) in the local ecology of (pre-existing) humans and non-humans (things). They identify, communicate with, relate to and interact thereafter with body external as well as embodied actants (humans and non-humans) in order to learn how the ideas/knowledge object may be materialized in their specific local setting. They try to design and construct new relations and interactions between people, things and their local context which have the outcome effects they pursue. They develop symbolic and socio-material tools during the process that help them with this, just as they work with overcoming the relational inertia that hinders the forming of a token-related performative actor network that consists of humans, certain types of interactions and performances, supporting artifacts and a narrative about the assembly that explains it. The outcome of the translation process may be that

1. A new assembly/an actor network (of humans and things) that—through their collective work—realizes the token (knowledge object) is constructed.
2. The assembly/actor network remains dynamically stable and thus keeps producing its local outcome effects over time.
3. A part of the assembly/actor network changes whereby the outcome effects of the assembly change
4. The relations and interactions between the humans and non-humans (objects) in the assembly dissolve whereby the effects of the token/ideas/knowledge object cease to exist in the organization.

#### 3.3.1. Implications and conditional propositions

So, what are the implications of the idea-practice translation model for the implementation of evidence-based knowledge objects in healthcare organizations? The idea-practice translation model assumes that a change like the introduction of an evidence-based knowledge object (a Cochrane review or guideline) will take place in an ecology of locally already existing humans and non-humans where some of them may be relevant to realizing/materializing the knowledge object/its ideas while others will not (3). The translator (perhaps a doctor) who wants to translate the knowledge object will bring his/her experiences from similar situations and their “life history” with them into the situation as well as their ideas about what the future goals are with introducing the knowledge object. They will “draw in” actants from these experiences which they assume are relevant in relation to translating (implementing) the knowledge object into their local context. These may concern humans or non-humans that according to their experiences may be relevant to implementing/translating the knowledge object, they may concern reflections about what may influence wished-for future states when introducing the knowledge object or personal experiences or feelings that the translator has about what may be needed or what may be a barrier to the introduction of the knowledge object in their specific context. As the translator(s) starts to implement the knowledge object, all the above-mentioned types of (unique) experiences will make him/her speak (ventriloquize them) in certain ways about what is needed to implement the knowledge object.

But other things will influence and make him/her speak, too. As the translator starts introducing the knowledge object, he/she will start communicating, interacting with and start trying to design and establish new types of relations and interactions between local humans and non-humans that the translator (according to his/her experiences) thinks are relevant to translating (implementing) the knowledge object. He/she may learn through this interaction process that some of them are indeed relevant to implementing (translating) the knowledge object and may be related and made start interacting in the way that the translator assumes. He/she may, however, also experience and learn that some of these humans and non-humans may not be related and made to interact in the necessary way and this will make him/her speak about these things (with other humans; doctors, employees etc.). Humans and non-humans (objects) not foreseen as relevant to the implementation of the knowledge object may also be “empirically” experienced to be relevant in unforeseen ways which will make the translator(s) speak about them; Rules and regulations may unexpectedly turn out to be in conflict with ideas presented in the knowledge object, the ideas about treatment of patients presented in the knowledge object may not fit the needs of all but only a certain group of the targeted hip replacement patients, economic restraints may make certain parts of the suggested treatment difficult because of limited economic resources in the department etc.).

All the controversies (difficulties) that the translator(s) experience with all these humans and non-humans are labelled “relational inertia” in the idea-practice translation model. Relational inertia is defined as “the accumulated and combined effect of conflicts and controversies that a translator meets and has to overcome as he/she tries to mobilize and assemble an actor network of humans and nonhumans making it possible to perform and thus realize a given innovative change idea and its related supposed and intended effects in an organization” (3). The relational inertia and controversies related to translating the knowledge object need to be overcome using whatever strategy or type of intervention that the translator finds necessary to do so. Symbol-based tools such as those developed by implementation science researchers in their theories, models and frameworks or theories and models developed in organizational change management research may be used to overcome controversies that hinder translation (implementation) of the knowledge object. Translators may also use local experiments as tools, too, to try out which relevant local humans and non-humans may or may not be related and made to interact in “wished-for ways” so that the knowledge object/its ideas may be realized.

Through this socio-material translation, design, construction and learning process the translator(s) gradually learn which humans and non-humans may or may not be relevant to translating (implementing) the knowledge object in his/her local context/ecology of humans and non-humans. If he/she succeeds the knowledge object is translated into a local narrative about what the knowledge object “looks” like in our department which includes certain people, certain types of interactions between them and types of performances by them as well as certain types of interactions with supporting artifacts. The outcome may be a narrative that states that “in our department the operation of Arthroplasties for proximal femoral fractures in adults should involve these actors (humans), who interact in this way following these procedures, using these prostheses and this type of cement (non-humans) based on these reasons” etc. Here an important point is that this narrative does not just represent an interpretation but is literally a representation of the performative actor network of humans and non-humans that was designed, constructed and made to interact and thus do “work” this way through the translation process in this specific department. This “assembly” of these humans, non-humans and the narrative about them may now be produced and reproduced through time and be stable, elements which are a part of the assembly may be changed whereby the assembly and its effects change, or the assembly may be dissolved whereby the knowledge object/its ideas cease to exist in the department.

The conditional propositions that may be derived from the idea-practice translation model are that: (1) A knowledge object in healthcare will not move by itself but will depend on translators doing the translation work necessary to make it move, (2) implementation of knowledge objects depends on local translators’ ability to interact, communicate with and learn from their interactions with their own experiences as well as locally present humans and non-humans, (3) it will moreover depend on translators’ ability to—through appropriate strategies/interventions, tools and handling of relational inertia—assure that an actor network of humans and non-humans doing the work realizing/materializing the knowledge object is established, (4) if successful, the outcome of the translation of the knowledge object/its ideas will be a performative actor network that consists of humans, certain types of interactions and performances, supporting artifacts and a narrative about the assembly that explains it.

### 3.4. The consequences of knowledge objects as travelling objects

At the time when organizational institutionalism emerged as a research stream in organization studies, organization theory was dominated by rational choice theory. Rational choice theory assumed that organizational change originated from bounded rational actors who adopted new management ideas, practices, and organizational forms because they wanted to make their organizations more efficient (71). However, research in neo-institutional theory showed that instead of being only rational, actors in organizations also adopted ideas, practices and organizational forms because they were embedded in social networks in institutional fields that—at a given point in time—considered these particular ideas, practices and organizing forms as legitimate (23, 72). Management ideas, practices and organizational forms were thus not just adopted by managers because they were rational but many times also because they gave these managers legitimacy in the eyes of other network participants.

Scandinavian neo-institutionalists (5) developed the “travel-of-ideas model” to theorize and model how management ideas, practices and organizational forms travel from the organizational field level and into and become institutionalized in local organizations/organizational units. It also theorized and modelled how innovative ideas, practices and organizational forms travelled the other way, that is from an organizational unit/an organization and out into the organizational field (see Figure 4).

Czarniawska and Joerges (5) adopted Latour's (4) concept of translation as the key concept to describe how these types of tokens were moved and travelled in and between organizations. As pointed out by Wedlin and Sahlin (50) when summing up the evidence about “the circulation of management ideas” in organization studies it is emphasized that it is a key insight from this research that “not only are ideas subject to translation as they are being circulated, but these ideas also have an impact on other ideas and on those organizations involved in the diffusion and adoption of ideas. Hence, the translation of ideas and their embeddedness in organizational practices and actions should be understood as sets of dynamic and mutually influencing processes” (50).

According to neo-institutional translation researchers tokens/knowledge objects thus move in time and space when they are translated, or as they choose to phrase it; “they travel” (5). This may seem odd since we are used to talking about humans as travelling but not tokens like ideas and objects etc. But again—if we look closer—it becomes apparent that tokens like knowledge objects do indeed travel. As an example, the organizational healthcare field in Denmark includes these actors: The ministry of Health, The Danish Health Authority, the 5 regions, local councils, hospitals, general practitioners, the medical industry, The Danish Medical Association, medical societies in different specialties, Cochrane Denmark, patients and their interest organizations. If a new Cochrane review of Arthroplasties (with and without bone cement) for proximal femoral fractures in adults (64) has been produced by Cochrane, it needs to travel between some of these actors to be implemented. The review may travel through different communication channels: two-way dialogues among people or mass-media channels. It may be communicated through articles in the magazine of the Danish Medical Association, through presentations at a conference or a seminar or it may be communicated through information letters by a medical society or through new regulations presented by the Danish Health Authority referring to the knowledge object. As a consequence, in all of these situations the token/knowledge object needs to move and “travel” between people and/or groups of people in organizations to be implemented.

The neo-institutional organization researchers Czarniawska and Joerges (5) studied how management ideas travel in and between organizations in organizational fields where human actors construct each other as belonging to the same field (as in the healthcare field mentioned above) (23). In their “travel-of-ideas model” they offer the following understanding of a successful translation (implementation) process in organizations:

1. An idea is firstly selected and attended to in moment/place A?? (a person or group notices the new Cochrane review that contains new ideas about treating a certain type of patient)
2. The ideas are then translated into an object (a text, a picture, a presentation or a prototype that explains how these ideas may be realized in “our” department) which is then translated into
3. New types of actions derived from the ideas that are then repeated and stabilized into an institution (a pattern of interactions that persists and continuously produces and reproduces and thus “materializes” the ideas related to the review across time and space).

The researchers (5) suggest that the way ideas travel between organizations in organizational fields is that “objectified ideas” (that is ideas that have been described as objects—perhaps a text or a PowerPoint presentation as suggested under point 2 above) are dis-embedded from the local context in the organization it comes from (for instance Cochrane) and are then later re-embedded in a local context of the receiving organization (hospitals,and other organizations and institutions in the healthcare field). A Cochrane review of Arthroplasties (with and without bone cement) for proximal femoral fractures in adults may thus arrive at a hospital through the field-related social networks within which the hospital is embedded whose employees then translate it into a clinical guideline which then starts travelling to other hospitals through field-related social networks, where it then becomes translated into new forms of actions, that then, if repeated over time, become institutionalized.

However, Czarniawska and Joerges (5) and other neo-institutional organization researchers (51, 52) have suggested that other competing ideas to a travelling idea—for instance those related to how to make Arthroplasties in the knowledge object—may exist and influence the travel of these ideas. Thus, instead of translators being rational or bounded rational in their decision-making they may also be influenced by other things. They may be motivated to adopt and translate new ideas about how to treat patients because they become fashionable (perhaps among surgeons) (52), because they serve their own or other people's interests (51) (surgeons who don't want to mass-produce based on standards but want to protect their professional autonomy as well as expertise by doing operations “their way”) or because they correspond with someone's (perhaps their own) ideology, because they are forced to do so (by regulative pressures from authorities) (23) or because dominating and highly legitimate field actors (like the Danish Medical Association) or local innovators and opinion makers notice and start “speaking on behalf” of the ideas/the knowledge objects and make them the “legitimate ideas/objects” to adopt (53).

### 3.4.1. Implications and conditional propositions

Knowledge objects and the ideas they contain need to be translated into objects and actions in certain ways to make the impact and produce the effects that Cochrane researchers associate with them. As a consequence of what was mentioned above, however, neo-institutional organization researchers would expect that a loyal one-to-one translation of a Cochrane review (and guideline) and its treatment ideas in a local department in a hospital will be a rare and unusual case (that would need to be studied) rather than an expected outcome of the rational communication and implementation of it. Thus if a new review of Arthroplasties (with and without bone cement) for proximal femoral fractures in adults and a clinical guideline developed based on it has been produced by Cochrane, it is not at all certain that the potential receivers of this review/guideline and its ideas will notice it and do “the rational thing” and just implement it when it is communicated. If the review/guideline happens to be noticed, it may not “just be implemented” but rather find itself in competition with other ideas about how to treat patients that did not originate from Cochrane and that may influence the translation of it. What the outcome of that complex, multi-actor and geographically dispersed translation process will be may be considered an empirical question.

The conditional propositions that may be derived from the idea model and neo-institutional research are: (1) The knowledge object and the ideas it contains will be “materialized” (and thus implemented) by being translated into objects and actions by the local receivers of it where it may or may not become repeated over time and thereby institutionalized, (2) the knowledge object and the ideas related to it will probably be changed as it moves and is translated by people and groups of people in the social networks of the organizational healthcare field, (3) what will influence the direction and content of the translation of the knowledge object/its ideas may be difficult to foresee with certainty and may be considered an empirical question.

## 4. Relevance of translation theories and models for implementation science^4^

As explained by Scheuer (73), processes of organizational change may be studied using variance or process theories about organizational change (48). Variance theories use independent variables as necessary and sufficient causes of variation in dependent variables. It may for instance be suggested that more of *X* and more of *Y* produces more of *Z*. Process theories use the sequence of events, activities and choices situated in time as well as in space to tell a story which explains how outcomes came about: They did A and then B to get C (48). These two approaches to the analysis of organizational change may be associated with two different ontological views: being and becoming realism (74). Being-realism is a fundamental ontological posture which asserts that reality pre-exists independently of observation and as static, discrete, and identifiable “things”, “entities”, “events”, “generative mechanisms”, etc. In contrast becoming-realism gives primacy to a processual view of reality. How an “entity” “becomes” constitutes what that actual entity is so that the two descriptions of an entity are not independent. Its “being” is constituted by its “becoming” (74).

Often implementation science theories, models, and frameworks (40) seem to study organizational change processes using a variance theory approach. The emphasis is put on developing process models, theories and determinant and evaluation frameworks, which provide a detailed description of how implementation processes related to the implementation of evidence-based knowledge unfold (as for instance described in Graham et al.'s Knowledge-to-action model (75)^5^, what variables or factors may enable or be a barrier during the implementation process as in the CFIR framework (78) and how implementation success may be measured [as in the evaluation frameworks by Glasgow (79) and Proctor (80)]. It is identifying the key aspects of processes and variables that influence the implementation process that is focused on, and it is the predictive potential of these theories, models, and frameworks for researchers as well as practitioners wanting to implement something that give them their scientific and normative value.

In contrast, translation theories and models build on a process and becoming-realist view that uses the sequence of events, activities and choices by translators situated in time as well as in space to explain how outcomes of translation/implementation processes came about. It is assumed that what a token—for instance a knowledge object—becomes is constituted by its becoming—that is by the translation process it goes through. Translation theories and models thus build on the “minimal” assumption that tokens in organizations—including management ideas, concepts and, as assumed here, knowledge objects—do not move by themselves but need to be moved by people (as suggested in Latour's (4) definition of translation above). To implement something necessitates construction of new relations and interactions between people and (for some translation researchers) things/objects (non-humans) that then—through their collective work—may (or may not) realize/materialize the token (3).

As a consequence, to foresee in advance what general variables may influence the translation/implementation process as well as what may enable or be a barrier to it (as in implementation science theories, models and frameworks) is “downplayed” in translation theories and models while developing a better processual understanding of how tokens (as a knowledge object, ideas, concepts, etc.) move and become “powerful” through the process of translation is given more attention.

Translation theories and models may thus offer implementation science researchers a new understanding of implementation as translation processes and some preliminary conditioned propositions about translation/implementation processes from which hypotheses may be developed and tested in future studies. On the other hand, implementation science researchers may offer organizational translation researchers insights into general theories, models, frameworks, concepts and variables concerning processes, enablers and barriers to translation/implementation that may make it possible for translation researchers and practitioners to identify in more detail what specific variables happen or happened to influence a particular empirical implementation/translation process. A few examples where the translation perspective may contribute to further developing implementation science and where implementation science may contribute to further developing organizational translation studies may be provided:

The CFIR (Consolidated Framework for Implementation Research) (78) was designed as a deterministic framework with the aim of creating a `one-stop shop' for clearly labelled and defined theoretical constructs to describe contextual factors that may have an impact on implementation success; specifically barriers and facilitators outside the evidence-based intervention that may hinder or facilitate efforts to integrate sustained change into clinical practice. It is comprised of five major domains: innovation characteristics, outer setting, inner setting, as well as characteristics of individuals and process. The process domain is related to stakeholders' perceptions of the success of the planning that took place when implementing an innovation including whether a context/needs assessment was completed, action items were developed and an implementation timeline, and whether implementation goals were set. The theoretical constructs describing contextual factors and what may hinder or facilitate implementation may inform translation researchers and give them a better understanding of which factors might influence the direction of translation processes. Translation theories and models may offer an alternative to the process understanding of the CFIR framework that does not focus on implementers planning processes but suggests that researchers should instead empirically follow and document how what translators along the translation chain do with a token—for instance a knowledge object—affect how that token is implemented and what the effects of that token turn out to be. This approach would make it possible to document empirically not just which planning factors and variables, but also which other contextual factors pointed out by the CFIR framework empirical data showed influenced the implementation (translation) of the token and its outcome. Moreover, it would make it possible to identify other variables not foreseen by the CFIR framework that may also have influenced the process and its (implementation/translation) outcome.

The above-mentioned translation approach to analyzing processes may also be relevant for The Theoretical Domains Framework (which is a determinant framework). It implies a system approach to implementation where the system is understood as an integrated whole composed of not only the sum of its components but also the relationships among those components (40, 81). It thus describes five interdependent determinants that are hypothesised to influence implementation processes and their outcomes;

• Characteristics of the implementation object,
• Influences at the individual healthcare professional level
• Patient influences,
• Collective-level influences,
• Effectiveness of implementation strategies to support implementation.

The framework, however, does not provide an understanding of through which types of processes these determinants become connected and end up producing certain outcomes during the implementation process. Here the translation perspective and its theories and models may contribute to a better understanding of these issues as they imply that a researcher needs to track and document how an implementation object becomes (or does not become) implemented/translated through translators' construction of new relations and interactions between people and (for some translation researchers) things/objects (non-humans) in certain contexts that then—through their collective work—does or does not realize/materialize the token/implementation object (3). By empirically tracking and documenting the translation process in this way, it would be easier for implementation researchers to identify and specify in more detail which types of influences affected or did not affect the process and how these influences did or did not come to do so.

An adaptation may be defined as a change to the content or delivery of an evidence-based intervention (EBI) that is designed to tailor the intervention to the needs of a given context (82). Adaptation researchers in implementation science have developed different types of frameworks to describe and identify the characteristics of adaptation processes; the Framework for Reporting Adaptations and Modifications -Expanded (FRAME) (83) and Moore et al.'s (84) framework. Kirk et al. (82) criticize these frameworks for only having a posthoc perspective which they consider shortsighted. They instead offer the “Adaptation-Impact Framework” which according to the researchers may be used to analyze the outcomes of adaptations after they have been finalized and implemented in the new context and used for proactive considerations of the potential impact of adaptations before they are finalized and implemented. The Adaptation-Impact Framework identifies three domains; (1) Adaptation characteristics which describe adaptations to the content and delivery as well as who delivered it and to whom, (2) possible Mediating or Moderating factors explaining why and how outcomes are achieved (through assuring fit and alignment of intervention with core components of intervention while considering the impact), (3) outcomes in relation to the intervention (client outcomes, service outcomes) and implementation (acceptability, appropriateness, adoption, feasibility, fidelity, cost, penetration, and sustainability) Kirk et al. (82) summarize the results of current adaptation research in this way:

“In general, research examining adaptation outcomes shows mixed results (85, 86): some adaptation efforts maintain or enhance outcomes of interest, whereas others diminish desired effects (87, 88) However, evidence is lacking regarding why and how adaptations produce demonstrated outcomes (that is, the pathways by which adaptations influence outcomes). Moreover, there is a lack of guidance and research on which outcomes (intervention or implementation outcomes) adaptations influence and how (that is, whether certain types of adaptations are more likely to influence certain types of outcomes, and what the total impact of adaptations will be” (82).

Here it may be suggested that using a process and becoming-realist translation view that uses the sequence of events, activities and choices by translators situated in time as well as in space to explain why and how adaptations produced certain outcomes seems highly relevant. It may be used to track and follow the pathway of translations through the translation chain making it possible to identify in more detail what influenced the direction and content of the adaptations that the translators made from point A to B and C etc. in time (and space).

Translation study researchers interested in how tokens change through translation processes may on their part learn a lot from the adaptation researchers in implementation science. Translation researchers in actor-network theory (4, 45, 46) suggest that both the token that moves (as an intervention or a knowledge object) and the humans that move it will change during the translation of it. Other translation researchers inspired by linguistic theory consider tokens (as interventions and knowledge objects) as texts (written or spoken) that need to be translated—ideally as loyal as possible—from a context A with one culture and language to another context B with another culture and language (7). Others try to theorize and model how such translation processes unfold as transfer, translation, and political negotiation processes (6) or complex socio-material design and construction processes (3). Here, however, implementation science adaptation researchers have a much richer vocabulary and several taxonomies describing which variables may influence and determine the content and direction of such processes that may be informative and contribute to translation researchers developing more refined perceptions of and ways to theorize these adaptive aspects of translation processes.

## 5. Conclusion

This article has discussed and identified some implications of organizational translation theories and models for processes related to implementation of evidence-based knowledge objects in healthcare organizations. It has also suggested some conditional propositions about translation of knowledge objects in such organizations that may be derived from them. It is concluded that organizational translation studies offer a new and different way of theorizing implementation processes in healthcare organizations. It is a way that assumes that the translation of tokens (including knowledge objects) unfolds through uninterrupted translation chains where the tokens need to be continuously given new energy and moved by people in a chain of translations to be implemented. The token will most likely be adjusted and changed through the translation process because the token and what counts as knowledge in relation to it will not just be transferred but also translated and politically negotiated as it moves. Finally, it may be concluded that in a translation view people and, according to some translation researchers, also objects/materials will need to be mobilized and influenced to act on behalf of a token (as a knowledge object) to translate and thus implement it in a local context.

However, to make people and objects act on behalf of and through those actions in practice “realize” a token is—as demonstrated—not easy. It depends on and demands that a lot of different and typically locally unique types of translation work is done before a token may be “implemented”. It may depend on translators' ability to introduce and adjust the token to a unique, pre-existing local context and ecology of humans and non-humans (objects/things) and it may depend on translators' ability to design and construct new relations and interactions between people and (for some translation researchers) things/objects (non-humans) that then—through their collective work—may (or may not) realize/materialize the token in the local context in focus (3) As a consequence the assumption that knowledge (ideas) may be stored in physical knowledge objects/texts which may then be transferred and reproduced by others/the receivers in an objective and thus non-subjective way seems questionable to translation study researchers. Moreover, they would suggest that the idea that the content of knowledge objects may be transferred from a sender to a receiver and may be implemented without being changed by the activities and translation processes of the actors involved seems if not unlikely then at least very uncertain.

It has been suggested that implementation science researchers seem to prefer a variance theory approach in their research that builds on a being-realist ontological posture where it is identifying the key aspects of processes and variables that influence implementation processes that are focused on and it is the predictive potential of these theories, models and frameworks for researchers as well as practitioners wanting to implement something that gives them their scientific and normative value. This was contrasted with the process and becoming-realist view of organizational translation study researchers who use process theories and the sequence of events, activities and choices situated in time as well as in space to tell a situated story about how outcomes of translation (implementation) processes came about. Here emphasis was ***not*** put on trying to theorize, model and foresee which variables may influence the change process in advance (as often seen in implementation science research) but on theorizing and modelling in more detail the process through which change comes about. The first approach suggests that a practitioner should build his/her implementation decisions on theories, models, and frameworks that general research evidence has shown influence implementation processes. The second approach proposes that the practitioner should focus on understanding the processes through which local changes come about but “downplay” his/her attempts to foresee in advance which other factors and variables may influence the content and direction of the (translation) process.

Consequently, it was suggested that translation theories and models may offer implementation science researchers a new understanding of implementation as translation processes and some preliminary conditioned propositions about translation/implementation processes from which hypotheses may be developed and tested in future studies. Implementation science researchers may on their part offer organizational translation researchers insights into general theories, models, frameworks, concepts and variables concerning processes, enablers and barriers to translation/implementation that may make it possible for translation researchers to identify in more detail what specific variables happen or happened to influence a particular empirical implementation/translation process in focus. Some examples of where implementation research may benefit from translation studies research and where translation studies research may benefit from implementation science research were provided.

## Contribution to the field

The translation perspective on organizational change have been developed in organization and management studies in recent years. Recent reviews of this perspective and research on organizational change have been written by (1–3). The contribution of the article is to demonstrate how organizational translation theories and models may offer implementation science a new perspective on the processes through which knowledge objects as Cochrane reviews, clinical guidelines and reference programs are implemented in practice in healthcare organizations and on what the difficulties may be in that connection. The article thus hypothesizes that findings, theories and models from organizational translation studies may also be relevant for implementation science researchers and practitioners. A hypothesis and empirical question that will however- as stated in the article -depend on further research to be answered.

## Data Availability

The original contributions presented in the study are included in the article, further inquiries can be directed to the corresponding author.

## Footnotes

^1^An example of such typologies is Demers (15) book “Organizational Change Theories” where she presents organization theories and the change strategies that may be derived from them using contingency theory (16–20), organizational life-cycle theory (21), population ecology theory (22), institutional theory (23), configurational theories (24, 25), organizational psychological theory (26, 27), organizational culture theory (28, 29) theories about organizations as political systems (30), behavioral and adaptive learning theory (31, 32), evolutionary theory (33, 34), complexity theory (35–37) and many more.

^2^That this is the case seems obvious when developing IT-systems in hospitals. IT-systems developed to monitor diabetes patients in their homes from the hospital thus include attempts to design and construct new relations between both humans (the patients, doctors and nurses supposed to monitor the patients) as well as non-humans (physical objects such as body-sensors, IT-modems, internet-connections, computers in the patients' homes as well as in the hospital, software systems, etc.). It moreover includes attempts to design and construct new types of relations and interactions between these humans and non-humans that may produce certain wished-for effects (a system that makes it possible to monitor and help diabetes patients with remembering to take their medicine and to keep them away from the expensive hospital beds).

^3^They are socio-technical/socio-material because they include and depend on both humans' and non-humans' (that is objects’) work.

^4^Since the author of this article is not an implementation science researcher and the aim of this article is not to compare the translation perspective on change with the perspectives of change found in implementation science, it is not relevant or possible to cover all the dimensions where the translation theories and models may be similar to or different from implementation science theories, models and frameworks. Instead, the aim of the article is to offer some insights and conditioned propositions based on theories and models from organizational translation studies that may offer implementation science researchers some new views on and, if further developed by implementation science researchers, hypotheses about what may characterize implementation processes in healthcare organizations. As a consequence, the following discussion will have a limited scope and only cover the relevance of translation theories and models for a few selected theories and frameworks in implementation science as seen from an organization studies researcher's point of view.

^5^Both knowledge translation and implementation science aim to bridge the gap between research and practice. Knowledge translation is about ensuring that knowledge users are aware of and use research findings in their decision-making. Implementation science studies the “black box” between research and practice to understand how evidence-based interventions can be successfully integrated into practice (76). However, implementation science and knowledge translation researchers do not seem to agree on a clear distinction between the two approaches. Nilsen (40) thus relates the knowledge-to-action model (75) to the process model category in his overview over theories, models and frameworks used in implementation science while the knowledge translation researchers Straus et al. (77) identify the same model as the key knowledge translation model in their introductory book: Knowledge Translation in Health Care - Moving from Evidence to Practice (Wiley Blackwell). It may therefore be suggested that the translation theories and models and the conditioned propositions derived from them in this article may be as relevant to knowledge translation researchers as they are to implementation science researchers. However, it should also be emphasized that whether that is the case will depend on further research.
